# Editorial: Natural Variations and Genetic Constraints on Plant Nutrition

**DOI:** 10.3389/fgene.2022.941118

**Published:** 2022-06-24

**Authors:** Fenglin Deng, Fanrong Zeng, Gareth J. Norton

**Affiliations:** ^1^ College of Agriculture, Yangtze University, Jingzhou, China; ^2^ School of Biological Sciences, University of Aberdeen, Aberdeen, United Kingdom

**Keywords:** plant nutrition, mineral elements, toxic metal(loid)s, natural variations, quantitative genetics

The healthy growth, development, and productivity of plants require large numbers of essential and beneficial elements, alongside the uptake of these elements, toxic metals and metalloids are also taken up and accumulated in the tissues of plants due to the imperfect selectivity of the transporters (Clemens and Ma, 2016; Huang et al., 2020; Deng et al., 2021). A proportion of the mineral elements taken up by roots is accumulated in the edible organs for human consumption, with the exact proportion being determined by the element in question, the plant species, and the environmental conditions. The lack of essential elements leads to retarded growth of plants, as well as hidden hunger for humans (Stanton et al., 2022). It is estimated that about 2 billion people worldwide suffer from deficiencies of iron (Fe) and zinc (Zn) in their diet (Huang et al., 2020). On the other hand, excess intake of toxic elements through the food chain results in great health risk (Zhao et al., 2022). For instance, populations that consume cadmium (Cd) or arsenic (As) contaminated grains have an increased cancer risk (Deng et al., 2018; Zhao and Wang, 2020). Therefore, there is an urgency to develop ideal crops with elevated levels of Fe and Zn, as well as reduced As and Cd in their edible tissues.

Both environmental factors and genetic mechanisms contribute to elemental accumulation in plants. During the last decades, great achievements have been made in identifying the critical transporters for the accumulation of various elements (Che et al., 2018; Huang et al., 2020; Yadav et al., 2021; Stanton et al., 2022; Zhao et al., 2022). Furthermore, the increasing datasets of genome sequencing and re-sequencing, pan-genomics, and RNA-seq provide abundant data resources to study the natural variations of critical genes responsible for mineral transport and accumulation in plants (Yang et al., 2018; Norton et al., 2019; Tan et al., 2020; Ruang-areerate et al., 2021). In this special issue, Liu et al. identified 167 unique and 25 repeatable quantitative trait loci (QTLs) in rice (*Oryza sativa* L.) affecting the concentration of 16 elements using 3,117 single nucleotide polymorphisms (SNPs), while only 40 QTLs were detected with 175 restriction fragment length polymorphism markers with the same population (Zhang et al., 2014).

This Research Topic focuses on the genetic conservation and variation of gene alleles responsible for the accumulation and detoxification of mineral element including both essential and toxic metal (loid)s in plants. Its ultimate aim is to identify the genetic regulators of mineral accumulation in plants, so as to exploit these mechanisms to both food security and safety. Cereals represent the most significant dietary intake of both essential and toxic minerals (Li et al., 2011; Deng et al., 2019; Huang et al., 2020). In this special issue, we include four original research articles covering aspects of both essential and toxic elements accumulation in rice and wheat (*Triticum aestivum* L.), which are the staple food for approximately 4.0 and 2.5 billion people (Gerard et al., 2020; Huang et al., 2020), respectively.

To gain nutritionally enhanced wheat with higher levels of Fe and Zn, a population consisting of 190 recombinant inbred lines (RILs) derived from a “Kachu” × “Zinc-Shakti” cross were generated. Classical QTL analysis revealed seven new pleiotropic QTLs for grain Zn and Fe accumulation, several candidate genes including putative transporters, regulators involved in ion binding, GTP or phospholipid binding were suggested to underlie these QTLs. The identified molecular markers and RILs with elevated grain Fe and Zn levels will be valuable for breeding wheat with increased concentrations of these key nutrients for people (Rathan et al.).

Apart from the classical approach to identify QTLs, candidate gene-based association analysis, genome-wide association study (GWAS), and multivariate QTL analyses have been employed for studying genetic variation of the rice ionome in this issue (Liu et al.; Liu et al.; Pinson et al.). Several key genes involved in Zn transport and accumulation in rice have been functionally characterized, to evaluate the effects of these genes related to grain Zn content, two panels of breeding lines and a multiparent advanced generation intercross (MAGIC) population were employed. Among the examined 65 candidate genes, a total of 8 genes were detected to be significantly associated with grain Zn concentration. The contribution to the variation was estimated to range from 7.70% to 17.99%. More importantly, *OsIRT1* and *OsZIP7* were identified from two of the three populations, indicating their broad variations and large effects (Liu et al.). The identified superior genes and haplotypes are excellent resources for further enhancement of Zn in rice grains.

To reveal the relationships among As-related traits, multiple GWA-QTL for hull silica (Si) concentration, grain concentrations of As, phosphorus (P), sulfur (S), calcium (Ca), and copper (Cu), as well as disease severity for straighthead disorder (StHD) (putatively attributed to As toxicity), were detected with in a rice minicore collection. A total of 195 QTLs for the examined nine traits were identified, six of the 15 QTLs which were detected for grain As had not been reported previously. The accumulation of Si, P, S, and Ca were not associated with grain-As at the QTL level. Unexpectedly, only four among of the 33 QTLs for StHD co-located with QTLs for grain As, additionally StHD severity did not correlate with grain As concentration. However, StHD QTLs did overlap with Si QTLs (11/33). The results indicate that the genetic factors regulating grain As and StHD are complex, and Si accumulation in plants is most likely to contribute to StHD severity (Pinson et al.).

Covaration of mineral elements is widely observed in plant tissues and is affected by environmental factors, however, traditional QTL analyses using individual elemental concentrations as separate traits, does not take this interrelatedness into consideration. To better understand the genetic basis of covariation of multiple elements, principle component analysis (PCA) of the examined traits of a rice RIL population under various conditions was conducted and with the PCs used in the QTL analyses. As a result, 53 QTLs controlling covariance among elemental concentrations within a single environment/tissue (PC-QTLs), and 152 QTLs which determined covariation among elements across environments/tissues (aPC-QTLs) were detected. In addition to the PC-QTLs matching the QTLs detected using single traits, some PC-specific QTLs were also identified, implicating novel clues for further analysis (Liu et al.).

The genetic markers, loci, candidate genes as well as superior haplotypes responsible for variation in mineral accumulation in rice and wheat reported in this issue are potentially beneficial for generating cereals rich in essential mineral elements but with less toxic elements in grain.

## References

[B1] Che J. Yamaji N. Ma J. F. (2018). Efficient and Flexible Uptake System for Mineral Elements in Plants. New Phytol. 219, 513–517. 10.1111/nph.15140 29633285

[B2] Clemens S. Ma J. F. (2016). Toxic Heavy Metal and Metalloid Accumulation in Crop Plants and Foods. Annu. Rev. Plant Biol. 67, 489–512. 10.1146/annurev-arplant-043015-112301 27128467

[B3] Deng F. Yamaji N. Ma J. F. Lee S.-K. Jeon J.-S. Martinoia E. (2018). Engineering Rice with Lower Grain Arsenic. Plant Biotechnol. J. 16, 1691–1699. 10.1111/pbi.12905 29479780 PMC6131421

[B17] Deng F. Yu M. Martinoia E. Songs W.-Y. (2019). Ideal Cereals With Lower Arsenic and Cadmium by Accurately Enhancing Vacuolar Sequestration Capacity. Front. Genet. 10, 322. 10.3389/fgene.2019.00322 31024630 PMC6467212

[B4] Deng F. Zeng F. Chen G. Feng X. Riaz A. Wu X. (2021). Metalloid Hazards: From Plant Molecular Evolution to Mitigation Strategies. J. Hazard. Mat. 409, 124495. 10.1016/j.jhazmat.2020.124495 33187800

[B5] Gerard G. S. Crespo-Herrera L. A. Crossa J. Mondal S. Velu G. Juliana P. (2020). Grain Yield Genetic Gains and Changes in Physiological Related Traits for CIMMYT's High Rainfall Wheat Screening Nursery Tested across International Environments. Field Crops Res. 249, 107742. 10.1016/j.fcr.2020.107742 32255898 PMC7079639

[B6] Huang S. Wang P. Yamaji N. Ma J. F. (2020). Plant Nutrition for Human Nutrition: Hints from Rice Research and Future Perspectives. Mol. Plant. 13, 825–835. 10.1016/j.molp.2020.05.007 32434072

[B16] Li G. Sun G. X. Williams P. N. Nunes L. Zhu Y. G. (2011). Inorganic Arsenic in Chinese Food and its Cancer Risk. Environ. Inter. 37, 12191225. 10.1016/j.envint.2011.05.007 21632110

[B7] Norton G. J. Travis A. J. Talukdar P. Hossain M. Islam M. R. Douglas A. (2019). Genetic Loci Regulating Arsenic Content in Rice Grains when Grown Flooded or under Alternative Wetting and Drying Irrigation. Rice 12, 54. 10.1186/s12284-019-0307-9 31332547 PMC6646650

[B8] Ruang-areerate P. Travis A. J. Pinson S. R. M. Tarpley L. Eizenga G. C. Guerinot M. L. (2021). Genome-wide Association Mapping for Grain Manganese in Rice (Oryza Sativa L.) Using a Multi-Experiment Approach. Heredity 126, 505–520. 10.1038/s41437-020-00390-w 33235293 PMC8026592

[B9] Stanton C. Sanders D. Krämer U. Podar D. (2022). Zinc in Plants: Integrating Homeostasis and Biofortification. Mol. Plant 15, 65–85. 10.1016/j.molp.2021.12.008 34952215

[B10] Tan Y. Sun L. Song Q. Mao D. Zhou J. Jiang Y. (2020). Genetic Architecture of Subspecies Divergence in Trace Mineral Accumulation and Elemental Correlations in the Rice Grain. Theor. Appl. Genet. 133, 529–545. 10.1007/s00122-019-03485-z 31734869

[B11] Yadav B. Jogawat A. LalNitrate S. K. Lakra N. Mehta S. Shabek N. (2021). Plant Mineral Transport Systems and the Potential for Crop Improvement. Planta 253, 45. 10.1007/s00425-020-03551-7 33483879

[B12] Yang M. Lu K. Zhao F.-J. Xie W. Ramakrishna P. Wang G. (2018). Genome-Wide Association Studies Reveal the Genetic Basis of Ionomic Variation in Rice. Plant Cell 30, 2720–2740. 10.1105/tpc.18.00375 30373760 PMC6305983

[B13] Zhang M. Pinson S. R. M. Tarpley L. Huang X.-Y. Lahner B. Yakubova E. (2014). Mapping and Validation of Quantitative Trait Loci Associated with Concentrations of 16 Elements in Unmilled Rice Grain. Theor. Appl. Genet. 127, 137–165. 10.1007/s00122-013-2207-5 24231918 PMC4544570

[B14] Zhao F.-J. Tang Z. Song J.-J. Huang X.-Y. Wang P. (2022). Toxic Metals and Metalloids: Uptake, Transport, Detoxification, Phytoremediation, and Crop Improvement for Safer Food. Mol. Plant 15, 27–44. 10.1016/j.molp.2021.09.016 34619329

[B15] Zhao F.-J. Wang P. (2020). Arsenic and Cadmium Accumulation in Rice and Mitigation Strategies. Plant Soil 446, 1–21. 10.1007/s11104-019-04374-6

